# Simvastatin Attenuates H_2_O_2_-Induced Endothelial Cell Dysfunction by Reducing Endoplasmic Reticulum Stress

**DOI:** 10.3390/molecules24091782

**Published:** 2019-05-08

**Authors:** Zhiqiang He, Xuanhong He, Menghan Liu, Lingyue Hua, Tian Wang, Qian Liu, Lai Chen, Nianlong Yan

**Affiliations:** 1 Department of Biochemistry and Molecular Biology, College of Basic Medical Science; Nanchang University, Nanchang 330006, China; hzq3231103954@163.com (Z.H.); hxhxhong@163.com (X.H.); 15797702796@163.com (M.L.); hly3288551238@163.com (L.H.); wt3508886849@163.com (T.W.); liucandice0412@163.com (Q.L.); 2 Laboratory Animal Research Center for Science and Technology, Jiangxi University of Traditional Chinese Medicine, Nanchang 330004, China; chlai3383@foxmail.com

**Keywords:** endothelial cell dysfunction, endoplasmic reticulum stress, simvastatin, Wnt/β-catenin pathway

## Abstract

Atherosclerosis is the pathological basis of cardiovascular disease, whilst endothelial dysfunction (ED) plays a primary role in the occurrence and development of atherosclerosis. Simvastatin has been shown to possess significant anti-atherosclerosis activity. In this study, we evaluated the protective effect of simvastatin on endothelial cells under oxidative stress and elucidated its underlying mechanisms. Simvastatin was found to attenuate H_2_O_2_-induced human umbilical vein endothelial cells (HUVECs) dysfunction and inhibit the Wnt/β-catenin pathway; however, when this pathway was activated by lithium chloride, endothelial dysfunction was clearly enhanced. Further investigation revealed that simvastatin did not alter the expression or phosphorylation of LRP6, but reduced intracellular cholesterol deposition and inhibited endoplasmic reticulum (ER) stress. Inducing ER stress with tunicamycin activated the Wnt/β-catenin pathway, whereas reducing ER stress with 4-phenylbutyric acid inhibited it. We hypothesize that simvastatin does not affect transmembrane signal transduction in the Wnt/β-catenin pathway, but inhibits ER stress by reducing intracellular cholesterol accumulation, which blocks intracellular signal transduction in the Wnt/β-catenin pathway and ameliorates endothelial dysfunction.

## 1. Introduction

Globally, the incidence and mortality rates of cardiovascular diseases remain very high [1]. Atherosclerosis (AS) is an important contributor towards the development cardiovascular and cerebrovascular diseases, whilst endothelial cell (EC) dysfunction plays a crucial role in the development of AS, since the overexpression of adhesion molecules and the release of inflammatory factors promote the formation of foam cells from macrophages [2]. Many studies have shown that oxidative stress is an important factor in EC dysfunction, as reactive oxygen species are small signaling molecules that play important roles in the regulation of biological processes and cellular functions [3]. When intracellular reactive oxygen species levels exceed the cell tolerance limit, the excess reactive oxygen species attack the mitochondrial membrane, leading to mitochondrial dysfunction and EC dysfunction [4,5]. Therefore, the mechanisms of oxidative stress-induced EC dysfunction urgently require further study.

Simvastatin is a common hypolipidemic drug that inhibits hydroxymethylglutaryl coenzyme A (HMG-COA) reductase activity and thus endogenous cholesterol synthesis [6]. Numerous studies have shown that simvastatin inhibits AS via various mechanisms. For example, simvastatin suppresses atherosclerotic lesion formation in ApoE KO mice by down-regulating CD36 and calpain-1, thus reducing inflammation [7]. In clinical trials, simvastatin significantly reduced the circulating levels of total cholesterol, LDL-C, VLDL-C, and LDL-C, which are considered risk factors for AS [8,9,10]. Although simvastatin is recognized for its anti-arterial AS effects, the specific mechanism underlying its effects on oxidative stress-induced EC dysfunction remains unclear.

The classical Wnt/β-catenin pathway is highly conserved and is involved in multiple physiological processes, including embryonic development and organ formation [11]. For example, knocking out the embryonic β-catenin gene in animal models has been shown to inhibit mesoderm formation [12]. Moreover, abnormal activation or inhibition of the Wnt/β-catenin pathway has been associated with the occurrence of various diseases, including cancers and Parkinson’s disease [11]. In recent years, an increasing number of reports have shown that this pathway is associated with EC dysfunction and AS [13,14]. For example, Wnt-5a protein expression was significantly up-regulated during AS development and was positively correlated with AS severity in animal experiments and human endarterectomy samples [15,16]. However, Ma et al. showed that the Wnt/β-catenin pathway can be suppressed by pigment epithelium-derived factor (PEDF), which reduces oxidative stress and endothelial injury [17]. Furthermore, our previous studies have shown that activating the Wnt/β-catenin pathway promotes H_2_O_2_-induced endothelial dysfunction [18].

The endoplasmic reticulum (ER) is an organelle found in eukaryotic cells. Hyperlipidemia, oxidative stress, and calcium imbalance can all disrupt ER homeostasis and lead to ER stress [19,20]. To restore ER homeostasis, the chaperone protein, GRP78, dissociates from PERK, IRE1, and ATF6, activating downstream signaling pathways which reduce cell inflammation and apoptosis [21,22]. ER stress is also involved in AS development; the upregulated expression of key ER stress molecules (GRP78, p-IRE1, ATF6, and CHOP) has been reported in the plaques of ApoE KO mice [23,24]. In addition, studies have shown that numerous atherogenic risk factors can activate ER stress in the initial stages of AS, thus contributing to EC dysfunction and the progression of AS [25,26].

Simvastatin, the Wnt/β-catenin pathway, and ER stress clearly affect AS development; however, the links between these factors and EC dysfunction are unclear. We hypothesized that simvastatin reduces H_2_O_2_-induced EC dysfunction via the Wnt/β-catenin pathway and ER stress. To investigate this, we treated human umbilical vein endothelial cells (HUVECs) with simvastatin and measured the subsequent levels of EC dysfunction, Wnt/β-catenin signaling, and ER stress.

## 2. Results

### 2.1. Effects of Simvastatin on the Viability of HUVECs

**Figure 1:**
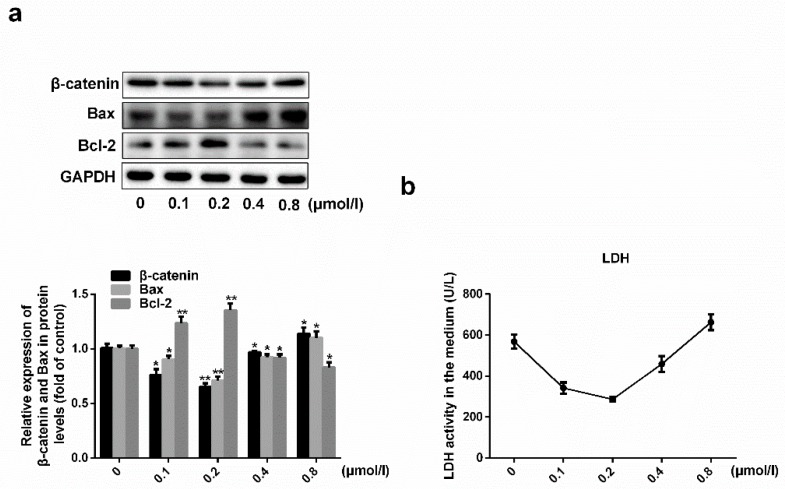
Effects of simvastatin on the viability of HUVECs. (**a**) The expressions of Bax, Bcl-2 and β-catenin in protein levels were analyzed by western blot analysis. (**b**) LDH level was measured. * *p* < 0.05 or ** *p* < 0.001 versus 0 group, *n* = 3.

To investigate the effects of simvastatin, HUVECs were exposed to different doses of the drug to determine its optimal concentration. As shown in Figure 1, increasing the concentration of simvastatin from 0 to 0.2 μmol/L decreased Bax and β-catenin expression and LDH levels in a dose-dependent manner and increased Bcl-2 expression. At simvastatin doses above 0.2 μmol/L, increasing the concentration increased Bax, β-catenin expression and LDH levels and decreased Bcl-2 expression. Therefore, the protective effect of simvastatin on HUVECs and its inhibition of Wnt/β-catenin signaling were most significant at 0.2 μmol/L (*p* < 0.001, *n* = 3) and this concentration was used in all future experiments.

### 2.2. Simvastatin Attenuates Oxidative Stress-Induced Endothelial Cell Dysfunction by Inhibiting the Wnt/β-Catenin Pathway

**Figure 2:**
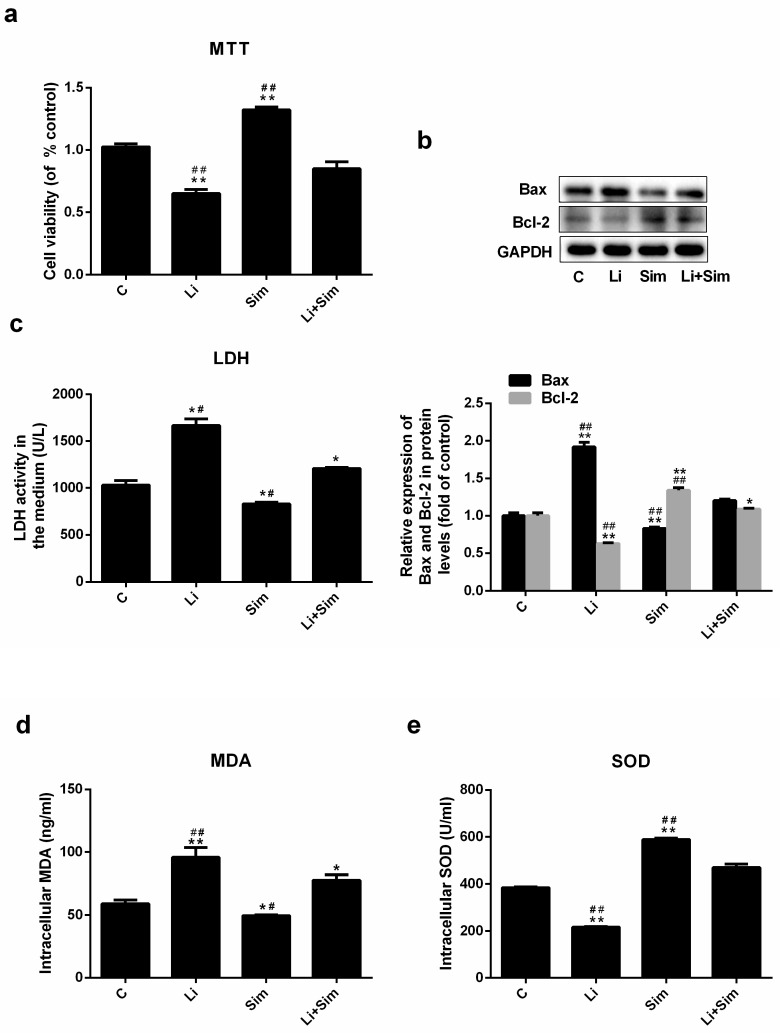
Simvastatin attenuates the dysfunction of endothelial cells under oxidative stress by inhibiting the Wnt/β-catenin pathway. (**a**) The viability of each group was measured by MTT assay (**b**) The protein levels of Bax and Bcl-2 (**c**) LDH level, (**d**) MDA content and (**e**) SOD activity were measured. * *p* < 0.05 or ** *p* < 0.001 versus C group; # *p* < 0.05 or ## *p* < 0.001 versus Li+Sim group, *n* = 3 or 6. C, HUVECs treated with H_2_O_2_; Li, C group treated with LiCl; Sim, C group treated with simvastatin; Li+Sim, C group treated with simvastatin and LiCl.

To evaluate the protective effect of simvastatin on HUVECs under oxidative stress, cell viability and the expression of Bax and Bcl-2 were measured. In the Li group, cell viability and Bcl-2 expression were 35% and 37% lower than that in the C group, respectively (Figure 2a), whilst in the Sim group, they were 32% and 34% higher than that in the C group, respectively. As expected, cell viability and Bcl-2 expression were lower in the Li+Sim group than in the Sim group (Figure 2a, *p* < 0.05, *n* = 3 or 6) but higher than in the Li group (Figure 2b, *p* < 0.001, *n* = 3 or 6), whilst the opposite result was observed for Bax expression (Figure 2b, *p* < 0.001, *n* = 3).

LDH levels in the medium and intracellular MDA concentration and SOD activity were also determined. LDH levels and MDA concentration were 23% and 35% higher in the Li group, and 20% and 38% lower in the Sim group than in the C group, respectively. LDH levels and MDA concentration in the Li+Sim group were lower than those in the Li group (Figure 2c, *p* < 0.05, *n* = 3) but higher than those in the Sim group (Figure 2d, *p* < 0.001, *n* = 3). SOD activity was 43% lower in the Li group and 53% higher in the Sim group than in the C group (Figure 2e, *p* < 0.001, *n* = 3), and was higher in the Li+Sim group than in the Li group, but lower in the Li+Sim group than in the Sim group (Figure 2e, *p* < 0.001, *n* = 3).

### 2.3. Simvastatin Reduces Endothelial Cell Adhesion by Inhibiting the Wnt/β-Catenin Pathway

**Figure 3:**
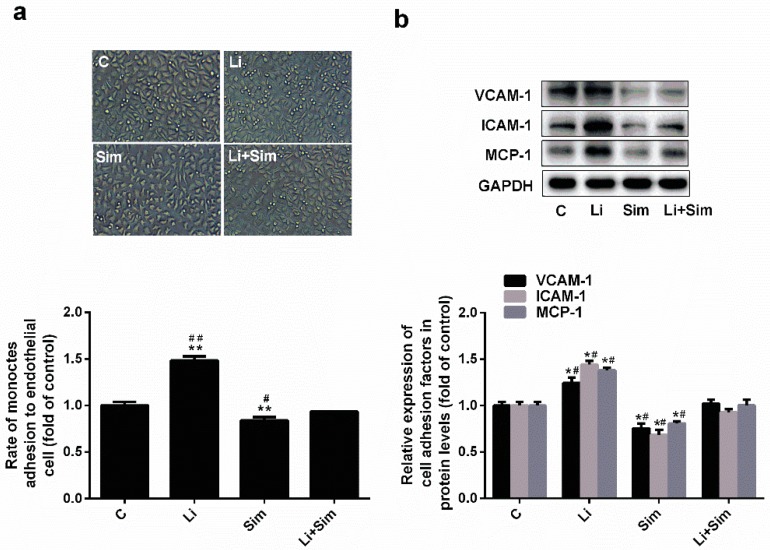
Simvastatin reduces the adhesion ability of HUVECs by inhibiting the Wnt/β-catenin pathway. (**a**) Adhesion rate of each group (**b**) Expressions of adhesion molecules in protein level. * *p* < 0.05 or ** *p* < 0.001 versus C group; # *p* < 0.05 or ## *p* < 0.001 versus Li+Sim group, *n* = 3. C, HUVECs treated with H_2_O_2_; Li, C group treated with LiCl; Sim, C group treated with simvastatin, Li+Sim, C group treated with simvastatin and LiCl.

The ability of the HUVECs to adhere to THP-1 cells was assessed by counting the number of THP-1 cells adhered to the HUVECs. As shown in Figure 3a, the number of THP-1 cells adhered to HUVECs in the Sim group was 16% lower than that in the C group (*p* < 0.001, *n* = 3). When the Wnt/β-catenin pathway was activated by LiCl, the adhesion ability of HUVECs increased by 48% (Figure 3a, *p* < 0.001, *n* = 3), and was higher in the Li+Sim group than in the Sim group yet lower than in the Li group (Figure 3a, *p* < 0.05 or 0.001, *n* = 3). The levels of adhesion molecule expression can affect the adhesion ability of HUVECs. As shown in Figure 3b, VCAM-1, ICAM-1, and MCP-1 expression was 24%, 31%, and 20% lower in the Sim group (*p* < 0.05, *n* = 3) and 25%, 44%, and 38% higher in the Li group (*p* < 0.05, *n* = 3) than in the C group, respectively. In the Li+Sim group, the expression of all of the adhesion molecules was lower and higher than that in the Li and Sim groups, respectively (Figure 3b, *p* < 0.05, *n* = 3). Taken together, these results indicate that simvastatin reduced the adhesion ability of HUVECs by inhibiting the Wnt/β-catenin pathway.

### 2.4. Simvastatin Inhibits ER Stress via the Wnt/β-Catenin Pathway

**Figure 4:**
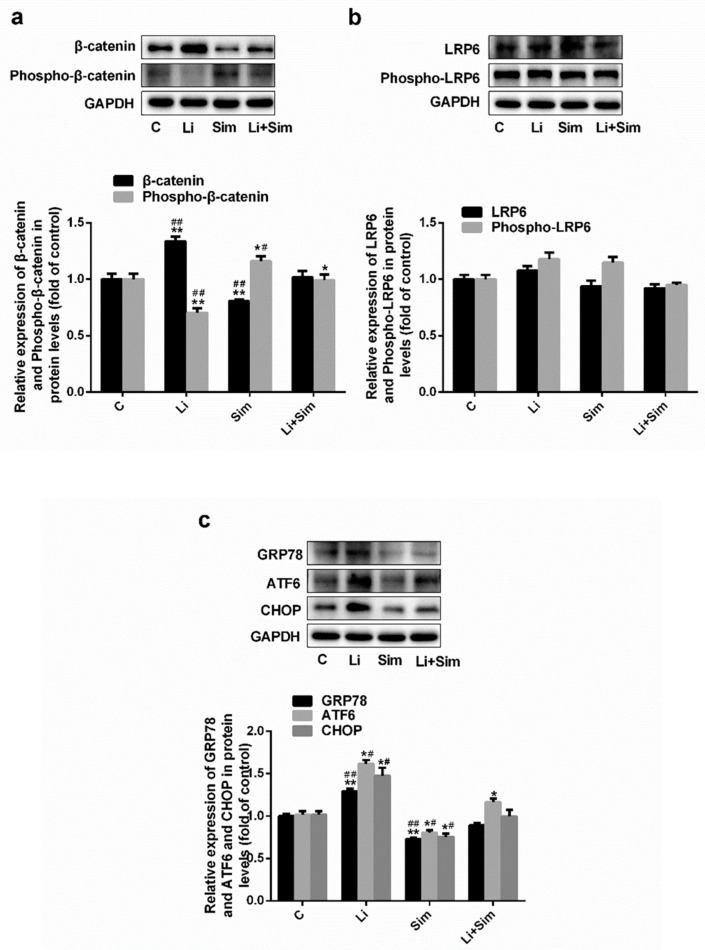
Simvastatin inhibits ER stress by inhibiting the Wnt/β-catenin pathway. The expressions of (**a**) β-catenin, phospho-β-catenin (**b**) LRP6, phospho-LRP6 (**c**) GRP78, ATF6 and CHOP in protein levels. * *p* < 0.05 or ** *p* < 0.001 versus C group; # *p* < 0.05 or ## *p* < 0.001 versus Li+Sim group, *n* = 3. C, HUVECs treated with H_2_O_2_; Li, C group treated with LiCl; Sim, C group treated with simvastatin; Li+Sim, C group treated with simvastatin and LiCl.

Under oxidative stress, simvastatin can reduce the injury and adhesion of HUVECs. To investigate the possible underlying mechanisms, the levels of β-catenin and phosphorylated β-catenin were determined by western blotting analysis (Figure 4a, *p* < 0.05 or 0.001, *n* = 3). β-catenin protein expression was 34% higher and 20% lower in the Li and Sim groups, respectively, compared to that in the C group. In addition, phospho-β-catenin levels 30% lower and 16% higher in the Li and Sim groups, respectively, compared to that in the C group. The levels of phosphorylated β-catenin in the Li+Sim group were lower than those in the Sim group but higher than those in the Li group.

LDL receptor-related protein 6 (LRP6) is a coreceptor of the Wnt/β-catenin pathway, and its phosphorylation activates the downstream Wnt/β-catenin pathway [27]. Lipid rafts can affect transmembrane signaling transduction in the Wnt/β-catenin signaling pathway. To determine whether simvastatin inhibits the Wnt/β-catenin pathway via lipid rafts, western blotting was performed to measure the levels of LRP6 and phosphorylated LRP6. As shown in Figure 4b, their levels in the Li and Sim groups did not significantly differ from those in the control group (*p* > 0.05, *n* = 3). Likewise, total and phosphorylated LRP6 levels in the Li+Sim group were not significantly different from those in either the Li or Sim groups (Figure 4b, *p* > 0.05, *n* = 3).

GRP78, ATF6, and CHOP are three key molecules in ER stress. In the Li group, GRP78, ATF6, and CHOP expression was increased by 29%, 61%, and 47%, respectively, compared to that in the C group (Figure 4c, *p* < 0.05 or 0.001, *n* = 3), indicating that ER stress may be activated by the Wnt/β-catenin pathway. 

In addition, GRP78, ATF6, and CHOP expression levels in the Sim group were 27%, 19%, and 24% lower, respectively, than those in the C group (Figure 4c, *p* < 0.05 or 0.001, *n* = 3), whilst in the Li+Sim group, their expression was significantly lower than those in the Li group, but higher than those in the Sim group (Figure 4c, *p* < 0.05 or 0.001, *n* = 3). These results indicate that simvastatin inhibits ER stress and that the Wnt/β-catenin pathway affects ER stress. 

### 2.5. Endoplasmic Reticulum Stress Affects the Wnt/β-Catenin Pathway

**Figure 5:**
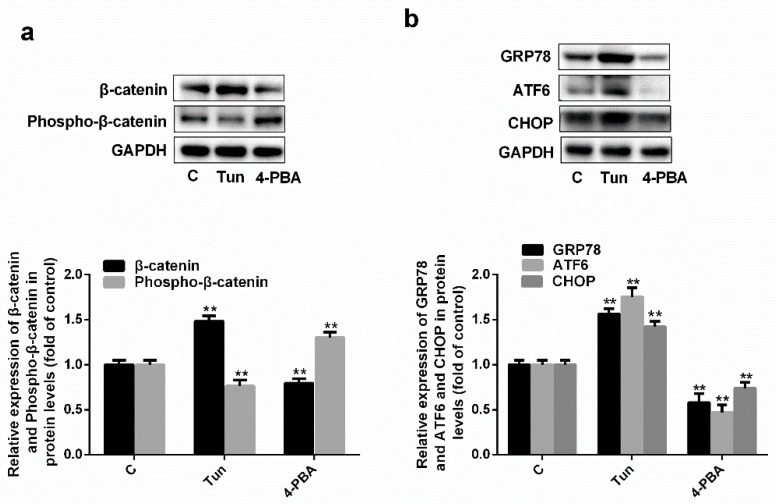
Endoplasmic reticulum stress can affect the Wnt/β-catenin pathway. Expressions of (**a**) β-catenin, phospho-β-catenin, (**b**) GRP78 ATF6 and CHOP in protein levels. ** *p* < 0.001 versus C group, *n* = 3. C, HUVECs treated with H_2_O_2_; Tun, C group treated with tunicamycin; 4-PBA, C group treated with 4-Phenylbutyric acid.

Tunicamycin is an ER stress activator, whereas 4-PBA is an ER stress inhibitor. As shown in Figure 5a, β-catenin expression increased by 48% and decreased by 20% after tunicamycin and 4-PBA treatment, respectively (*p* < 0.001, *n* = 3); however, phosphorylated β-catenin levels decreased by 24% and increased by 30% after tunicamycin and 4-PBA treatment, respectively (*p* < 0.001, *n* = 3). GRP78, ATF6, and CHOP expression increased by 58%, 76%, and 42%, respectively, after tunicamycin treatment, but decreased by 42%, 53%, and 26%, respectively, after 4-PBA treatment (Figure 5b, *p* < 0.001, *n* = 3). Thus, endoplasmic reticulum stress affects the Wnt/β-catenin pathway.

### 2.6. Simvastatin Reduces the Deposition of Cholesterol in the Cytoplasm

**Figure 6:**
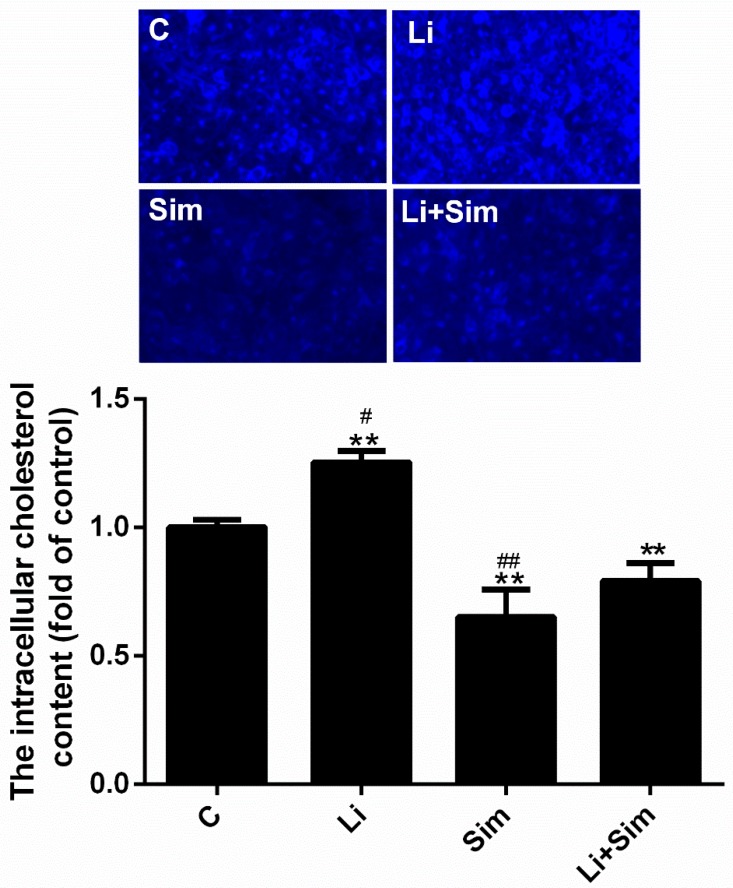
Simvastatin reduces the deposition of cholesterol in the cytoplasm. The intracellular cholesterol was measured by Filipin staining. ** *p* < 0.001 versus C group; # *p* < 0.05 or ## *p* < 0.001 versus Li+Sim group, *n* = 3. C, HUVECs treated with H_2_O_2_; Li, C group treated with LiCl; Sim, C group treated with simvastatin; Li+Sim, C group treated with simvastatin and LiCl.

To explore the possible mechanism underlying the inhibition of ER stress by simvastatin, intracellular cholesterol was measured by Filipin staining, as fluorescence intensity is proportional to intracellular cholesterol content. As shown in Figure 6, the cholesterol content was 25% higher in the Li group (*p* < 0.001, *n* = 3) and 35% lower in the Sim group (*p* < 0.001, *n* = 3) than in the C group, whilst it was significantly lower in the Li+Sim group than that in the Li group and higher than that in the Sim group (*p* < 0.05 or 0.001, *n* = 3).

## 3. Discussion

Simvastatin treatment reduces the risk of cardiovascular events and cardiovascular disease-related deaths, predominantly by decreasing intracellular and circulating cholesterol levels [7,8,9,10]; however, the role of simvastatin in ECs remains unclear. In this study, we found that low simvastatin doses increased the viability of HUVECs while high doses reduced their viability. Moreover, we found that low-dose simvastatin treatment blocked the Wnt/β-catenin pathway, whereas higher doses re-activated the pathway (Figure 1). Previous studies have shown that the activation of this pathway induces EC dysfunction [17,18]. These results suggest that low-dose simvastatin treatment may reduce HUVEC dysfunction by inhibiting the Wnt/β-catenin pathway.

To determine whether simvastatin could reduce Wnt/β-catenin pathway-induced EC dysfunction, LiCl was used to activate this pathway in HUVECs. LiCl inhibited the phosphorylation of cytoplasmic β-catenin, confirming Wnt/β-catenin pathway activation (Figure 4a). Reduced β-catenin phosphorylation prevents its degradation, leading to cytoplasmic accumulation and translocation into the nucleus. As expected, LiCl increased EC dysfunction (Figure 2 and Figure 3), further confirming that simvastatin regulates the Wnt/β-catenin pathway; we propose that simvastatin exerts a protective effect by inhibiting the Wnt/β-catenin pathway via β-catenin phosphorylation (Figure 4), leading to its degradation and reduced nuclear translocation.

Cholesterol is one of the main components of lipid rafts, which are involved in numerous cellular and signal transduction processes since many receptors and signal transduction-related proteins are localized in or associated with them [28]. For example, transmembrane signal transduction via the LPS receptor (TLR4) and TGF-β receptors is associated with and dependent upon lipid rafts [29,30]. LRP6 plays a vital role in mediating Wnt/β-catenin signal transduction, and its distribution in lipid rafts facilitates Wnt/β-catenin signaling [31,32,33]. Methyl-β-cyclodextrin can downregulate the expression of LRP6 and β-catenin by depleting cholesterol levels and disrupting the structure of lipid rafts. Furthermore, increased cholesterol levels increase clonogenic potential and upregulate the expression of LRP6 and β-catenin [34]. In this study, simvastatin was used to inhibit cholesterol biosynthesis. Figure 6 shows that intracellular cholesterol levels were decreased by simvastatin treatment; however, the expression and phosphorylation of LRP6 were not significantly affected (Figure 4b). These results suggest that simvastatin did not affect signaling upstream of the Wnt/β-catenin pathway.

ER stress occurs when the morphology and function of the ER are altered as a result of endogenous or exogenous stimuli, and may lead to the accumulation of misfolded proteins in the ER. During ER stress, the expression of GRP78, CHOP, and ATF6 increases [21]. Recent studies have shown that the Wnt/β-catenin pathway can affect ER stress; in cancer cells, inhibition of the Wnt/β-catenin pathway induces ER stress, which then leads to apoptosis [35,36]. Furthermore, Zhang et al. proposed that inhibiting the Wnt/β-catenin pathway and β-catenin degradation reverses the inhibition of ATF6 by LEF1, resulting in ATF6-induced ER stress in preadipocytes [37]. This pathway is negatively correlated with ER stress in preadipocytes; however, we observed contradictory results in HUVECs. Simvastatin blocked the activation of ER stress and the Wnt/β-catenin pathway while LiCl activated the pathway and increased the expression of ER stress-related proteins (ATF6, GRP78, and CHOP; Figure 4c), indicating that the Wnt/β-catenin pathway is positively correlated with ER stress in HUVECs. Although these results contradict one another, the Wnt/β-catenin pathway is clearly an important regulator of ER stress. In this study, simvastatin did not block transmembrane signal transduction in the Wnt/β-catenin pathway as it did not alter the expression or phosphorylation of LRP6 (Figure 4b), indicating that the Wnt/β-catenin pathway did not inhibit ER stress. Some studies believed that changes in ER lipid composition under cholesterol overload may disrupt ER membrane functions and cause an accumulation of misfolded and unfolded ER proteins, which can trigger ER stress [38,39,40,41]. Therefore, we propose that simvastatin blocks ER stress by inhibiting HMG-COA reductase, thus reducing intracellular cholesterol accumulation in HUVECs (Figure 6).

To further investigate the mechanism underlying the inhibition of the Wnt/β-catenin pathway by simvastatin, HUVECs were treated with tunicamycin (an ER stress activator) or 4-PBA (an ER stress inhibitor). When ER stress was induced by tunicamycin, total β-catenin levels increased and phosphorylated β-catenin levels decreased. When ER stress was inhibited by 4-PBA, total β-catenin levels decreased and phosphorylated β-catenin levels increased (Figure 5). Therefore, we concluded that simvastatin inhibited the Wnt/β-catenin pathway by inhibiting ER stress, since simvastatin did not affect the expression or phosphorylation of LRP6.

**Figure 7:**
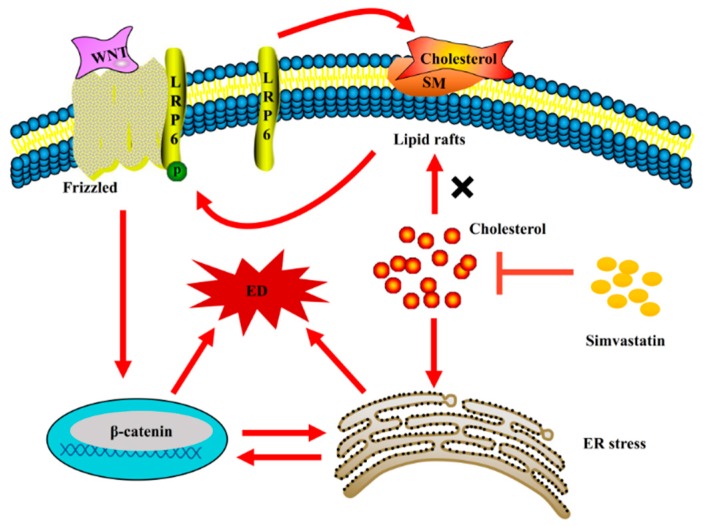
The mechanism of simvastatin in alleviating endothelial dysfunction under oxidative stress. LRP6 is localized to the cell membrane, and it is phosphorylated after it accumulates in the lipid raft which contributes to activate intracellular signal transduction in the Wnt/β-catenin pathway and then induce ER stress and endothelial dysfunction. Simvastatin can block intracellular cholesterol synthesis, which depresses endoplasmic reticulum stress and endothelial dysfunction, but does not further affect the phosphorylation of LRP6 by affecting lipid rafts. Therefore, although, simvastatin does not affect transmembrane signal transduction in the Wnt/β-catenin pathway, but blocks intracellular signal transduction in the Wnt/β-catenin pathway by attenuating the ER stress.

Altogether, our results show that simvastatin did not affect transmembrane signal transduction in the Wnt/β-catenin pathway, but inhibited ER stress by reducing intracellular cholesterol accumulation, which blocked intracellular signal transduction in the Wnt/β-catenin pathway and reduced endothelial dysfunction (Figure 7).

## 4. Materials and Methods

### 4.1. Cell Culture and Treatment

HUVECs were purchased from the Cell Bank of the Type Culture Collection of the Chinese Academy of Sciences (Shanghai, China) and were cultured in DMEM containing penicillin (100 U/mL), streptomycin (0.1 mg/mL), and 10% fetal calf serum at 37 °C in an incubator containing 5% CO_2_. THP-1 cells (Cell Bank of the Type Culture Collection of the Chinese Academy of Sciences) were cultured in RPMI-1640 and incubated under the same conditions as the HUVECs.

### 4.2. Grouping and Dosing

To determine the optimal simvastatin concentration, HUVECs were cultured in 6-well plates. When the cells reached 70% confluence they were treated with different doses of simvastatin (0, 0.1, 0.2, 0.4, or 0.8 μmol/L) for 24 h. The lactate dehydrogenase (LDH) level in the medium and β-catenin, Bax, and Bcl-2 protein levels were then quantified. To determine whether simvastatin had a protective effect on endothelial cells under oxidative stress and to identify the underlying mechanism, HUVECs were treated with either lithium chloride (LiCl), simvastatin, or both. HUVECs were divided into the following four groups: C, treated with H_2_O_2_; Li, treated with LiCl + H_2_O_2_; Sim, treated with simvastatin + H_2_O_2_; and Li+Sim, treated with simvastatin + LiCl + H_2_O_2_. After cells reached 70% confluence, simvastatin (0.2 μmol/L) was added to the Sim and Li+Sim groups. Two hours later, LiCl (20 μmol/L) was added to the Li and Li+Sim groups. After 24 h, 500 μmol/L H_2_O_2_ was added to the C, Li, Sim, and Li+Sim groups. Then after another 24 h, the cells were collected for further analysis. To investigate whether the Wnt/β-catenin pathway affected ER stress, HUVECs were treated with tunicamycin (10 μmol/L, Tun group) or 4-phenylbutyric acid (10 mmol/L, 4-PBA group). The protein expression of β-catenin, phospho-β-catenin, and the ER stress-related molecules, GRP78, ATF6, and CHOP, was measured.

### 4.3. Measurement of LDH Levels in the Medium and Intracellular SOD Activity and MDA Concentration

The LDH level in the culture medium and the intracellular SOD activity and MDA concentration can reflect EC dysfunction. After the HUVECs were treated as described above, the medium was collected from the six-well plate and its LDH level was measured using a commercially available kit. The cells from the six-well plate were digested with trypsin, collected, disrupted using ultrasound, and centrifuged at 10,000 rpm for 10 min at 4 °C. The SOD activity and MDA concentration of the resulting supernatant were measured using a commercially available kit according to the manufacturer’s instructions.

### 4.4. Measurement of Adhesion Ability of HUVECs to THP-1 Cells

HUVECs were cultured in 24-well plates as described above. After incubation with H_2_O_2_ for 24 h, THP-1 cells (1 × 10^5^ cells/well) were added. After 2 h, the medium in each well was removed and unattached THP-1 cells were washed away with PBS. The number of THP-1 cells adhered to the HUVECs was counted using a microscope (magnification, ×20; Olympus IX71; Olympus Corporation, Tokyo, Japan).

### 4.5. Western Blotting Analysis

To measure protein expression levels, cells were lysed and total protein was collected. Samples were then boiled in protein loading buffer at 100 °C for 5 min. Total proteins were extracted from the cells, subjected to 8–12% SDS-PAGE, and transferred to polyvinyl fluoride microporous membranes. Membranes were blocked with 5% skim milk or 5% bovine serum albumin, dissolved in Tris-buffered saline/Tween 20 (TBST) for 1 h, and incubated at 4 °C overnight with primary antibodies (human apoptosis-associated proteins Bcl-2 and Bax; adhesion-associated proteins ICAM-1, VCAM-1 and MCP-1; Wnt/β-catenin pathway-associated proteins β-catenin, phosphorylated β-catenin, LRP6, and phosphorylated LRP6; key molecules of ER stress GRP78, ATF6, and CHOP; and GAPDH) at a 1:1000 dilution. Membranes were then washed three times with TBST and incubated with a secondary antibody for 1 h at room temperature. After washing three times, an enhanced chemiluminescent reagent was added to the membrane and the blot was visualized using an autoradiography system (Chemiluminescence Imaging System; version 5.1; Bio-Rad Laboratories, Inc., Hercules, CA, USA). Each set of experiments was performed three times. 

### 4.6. Measurement of Cell Viability by MTT Assay

To determine the effect of simvastatin on the viability of HUVECs under oxidative stress, they were seeded onto 96-well plates at a density of 1 × 10^5^ cells/well and cultured as described above. Next, 20 µL of MTT (5 mg/mL) was added to each well, the cells were incubated at 37 °C for 4 h, the medium was removed, and 200 µL of dimethyl sulfoxide was added to each well to dissolve the blue precipitate. The absorbance was measured at 490 nm using a microplate reader (Thermo Fisher Scientific, Inc., Waltham, MA, USA). A total of 6 wells were measured per group and the experiment was performed three times.

### 4.7. Filipin Staining

2.5 × 10^4^ cells were cultured in the 24-well plate after the glass lips were added. When HUVECs reached 70% confluence in the 24-well plate, the four cell groups were treated with different drugs, as described above. The medium in each well was then removed and the cells were washed three times with PBS. After being fixed with 4% paraformaldehyde for 30 min, the cells were washed with PBS three more times, 1.5 mg/mL of glycine was added to each well, and the cells were incubated in the glycine solution for 10 min. Finally, 500 μL of Filipin (0.05 mg/mL Filipin in PBS) was added to each well, incubated for 2 h, then the fluorescence intensity of each group was observed using a fluorescence microscope (magnification, ×10; Olympus IX71). The experiment was performed three times [42].

### 4.8. Statistical Analysis

The data were analyzed using the statistical software, SPSS 22.0 (IBM Corp., Armonk, NY, USA). Significant differences between the groups were determined using one-way analysis of variance. All results represent at least 3 independent experiments. Differences with *p* values < 0.05 were considered statistically significant.

## 5. Conclusions

Simvastatin did not affect transmembrane signal transduction in the Wnt/β-catenin pathway, but inhibited ER stress by reducing intracellular cholesterol accumulation, which blocked intracellular signal transduction in the Wnt/β-catenin pathway and reduced endothelial dysfunction.
